# A Comparative Study of Gemcitabine-Cisplatin vs. Dose-Dense MVAC (Methotrexate, Vinblastine, Doxorubicin, and Cisplatin) as Neoadjuvant Chemotherapy for Muscle-Invasive Bladder Cancer: A Single-Institution Experience

**DOI:** 10.7759/cureus.85071

**Published:** 2025-05-30

**Authors:** Varun Goel, Arpit Jain, Dharmishtha A Basu, Nivedita Patnaik, Akanksha Jaju, Vineet Talwar, Sudhir Rawal

**Affiliations:** 1 Department of Medical Oncology, Rajiv Gandhi Cancer Institute and Research Centre, New Delhi, IND; 2 Department of Pathology, BLK-Max Super Speciality Hospital, New Delhi, IND; 3 Department of Pathology, ESIC (Employees' State Insurance Corporation) Hospital, Basaidarapur, New Delhi, IND; 4 Department of Urology, Rajiv Gandhi Cancer Institute and Research Centre, New Delhi, IND

**Keywords:** bladder cancer, chemotherapy, gemcitabine-cisplatin, mvac, neoadjuvant

## Abstract

Background

Muscle-invasive bladder cancer (MIBC) involves a high risk of progression and death. Neoadjuvant chemotherapy (NAC), prior to radical cystectomy (RC), has been shown to improve survival. Gemcitabine-cisplatin (GC) has emerged as a preferred NAC regimen based on its favorable toxicity profile, while dose-dense methotrexate, vinblastine, doxorubicin, and cisplatin (ddMVAC) may yield better pathologic complete response (pCR) rates, as reported in some studies.

Purpose

The objective of this study is to compare the efficacy and toxicity profiles of GC and ddMVAC as neoadjuvant regimens in patients with MIBC.

Methods

This was a retrospective study of 120 patients with clinical stage cT≥2-4a, N+M0 MIBC, treated at a single tertiary cancer center from January 2023 to December 2024. GC was administered to 63 patients, and ddMVAC to 57 patients. Outcomes included pCR, pathologic downstaging, disease-free survival (DFS), overall survival (OS), and toxicity.

Results

The pCR rate was higher for ddMVAC (22/57 patients, 38.59%) than for GC (14/63 patients, 22.22%) (p = 0.04). Patients with downstaging (≤pT1N0) were more frequent in the ddMVAC group (34/57 patients, 59.64%) compared to the GC group (28/63 patients, 44.44%). The median OS was longer for the ddMVAC group (41.6 months; 95% CI: 35.2-48.9) compared to the GC group (36.8 months; 95% CI: 30.5-42.1) (p = 0.12; HR: 0.82; 95% CI: 0.63-1.06). The incidence of grade ≥3 toxicity was higher for ddMVAC, with 20/57 patients (35%) vs. 10/63 patients (15.87%) for GC. Febrile neutropenia occurred in 8/57 patients (14%) in the ddMVAC group, compared to 3/63 patients (4.76%) in the GC group.

Conclusion

ddMVAC exhibited higher pCR and downstaging rates than GC, with a tolerable - though higher - rate of toxicity. Both regimens are acceptable options for NAC in MIBC, with treatment choice dependent on each patient’s fitness and preferences.

## Introduction

Muscle-invasive bladder cancer (MIBC), which represents approximately 25%-30% of newly diagnosed cases of urothelial carcinoma [1], is a clinically aggressive form of bladder cancer, defined by invasion of the muscularis propria of the bladder wall (T2 or higher). Although radical cystectomy (RC) is the conceptual anchor of definitive local treatment for bladder cancer, a considerable number of patients will experience relapse as distant metastases, with the poorest long-term survival. In fact, the five-year overall survival (OS) for patients treated with surgery alone is about 50%, indicating that additional systemic therapy is needed to control disease that has already disseminated as micrometastases to other parts of the body [2].

Neoadjuvant chemotherapy (NAC), given before RC, is now the standard of care for all eligible patients with MIBC. The rationale for NAC includes elimination of occult micrometastases, downstaging of the tumors, assessment of the patients’ chemosensitivity, and potentially setting the stage for bladder-sparing management strategies. Landmark studies, including SWOG-8710 and the Advanced Bladder Cancer (ABC) meta-analysis, showed that cisplatin-based NAC improved OS [2,3]. The studies showed a 5%-8% absolute increase in OS at five years. Two chemotherapy regimens dominate the NAC context: gemcitabine-cisplatin (GC) and dose-dense methotrexate, vinblastine, doxorubicin, and cisplatin (ddMVAC). GC, which was originally validated in the metastatic urothelial carcinoma population, has become preferred in the neoadjuvant space due to the improved toxicity profile and logistical considerations over ddMVAC [4,5]. ddMVAC is an abbreviated regimen of the traditional MVAC regimen, with shorter cycles and growth factor support, and has demonstrated greater response rates and better tolerability than standard MVAC in the advanced disease context [6,7]. Additionally, some prospective and retrospective studies have indicated higher pathologic complete response (pCR) rates with ddMVAC in the NAC context, which may lead to improved long-term outcomes [8-10]. Unfortunately, there is a lack of randomized, head-to-head comparisons of GC and ddMVAC in the NAC setting. 

Adjuvant immunotherapy holds great promise as well, with the CheckMate 274 trial reporting that nivolumab demonstrated improved disease-free survival (DFS) in high-risk MIBC patients post-surgery and NAC [11]. Cost-effectiveness studies, primarily based on VESPER trial data, have hypothesized that ddMVAC may be less costly than GC when estimated long-term oncologic outcomes are factored in [12-14]. Consequently, primary clinical guidelines, such as those of the National Comprehensive Cancer Network (NCCN), European Association of Urology (EAU), and American Society of Clinical Oncology (ASCO), accept cisplatin-based NAC for patients with good performance status and normal renal function [15].

A meta-analysis confirmed the survival advantage associated with NAC in MIBC and justified routinely considering this therapy in appropriate patients [16]. Due to ongoing differences of opinion related to the best NAC regimen, and the changing treatment environment of bladder cancer, real-world evidence regarding the effectiveness of these regimens in real-life populations is also invaluable. Furthermore, toxicity considerations - especially in older patients and/or those with borderline renal function - are still important factors in making regimen decisions. Although ddMVAC may be more efficacious, it may not be suitable for certain subpopulations due to side effects, including increased hematologic and gastrointestinal complications.

This single-center retrospective study was conducted to compare the efficacy and safety of GC and ddMVAC as neoadjuvant regimens for MIBC. We sought to identify differences in pCR rates, tumor downstaging, DFS and OS, and treatment-related toxicities across all groups. The intention of this retrospective study is to further clarify decision-making for oncologists and multidisciplinary teams in real-life clinical practice. Our aim was to contribute to this evidence by providing real-world data from a single institutional experience, particularly from a population in which such comparative analyses remain limited.

## Materials and methods

Study design and population

This retrospective cohort study was conducted at Rajiv Gandhi Cancer Institute and Research Centre, New Delhi, India. The study included patients with histologically confirmed urothelial carcinoma of the bladder, clinical stage cT≥2-4a, N+, M0, who received NAC followed by RC from January 2023 to December 2024. Patients were excluded from the study if they had non-urothelial histology or did not receive at least one cycle of the planned NAC. All included patients were deemed eligible for cisplatin-based therapy. The study period for patient inclusion was January 2023 to December 2024. The sample size was determined by the number of eligible patients treated with either GC or ddMVAC as NAC, followed by RC within this timeframe at our institution.

Treatment regimens

The GC group (n = 63) received gemcitabine 1000 mg/m² on days 1, 8, and 15, and cisplatin 70 mg/m² on day 1, every 28 days, for three to four cycles. The ddMVAC group (n = 57) received methotrexate 30 mg/m², vinblastine 3 mg/m², doxorubicin 30 mg/m², and cisplatin 70 mg/m² on day 1, every 14 days, for four cycles, along with granulocyte colony-stimulating factor (G-CSF) support. However, in this retrospective study, it is noted that chemotherapy doses and the number of cycles administered were sometimes adjusted at the treating physician's discretion, based on the patient's overall condition, comorbidities, and tolerance to treatment.

Treatment Assignment

Selection of treatment options between GC and ddMVAC was made by a multidisciplinary team, with consideration of patient performance status, renal function, comorbidities, and preference.

Adherence

Completion rates of planned chemotherapy cycles were 90.5% in the GC group, and 84.2% in the ddMVAC group. Reasons for discontinuation or delay included, but were not limited to, toxicity and patient choice.

Pathology Review

The cystectomy specimens were reviewed by dedicated genitourinary pathologists at our center. These were all done within the context of a standardized pathological assessment protocol, set according to the AJCC TNM 8th edition criteria.

Outcomes and assessment

The primary endpoint of this retrospective study was pCR, defined as ypT0N0 at cystectomy. Key secondary endpoints included pathological downstaging to ≤ypT1N0, OS, DFS, and documented adverse events (grade ≥3, according to CTCAE v5.0). Data regarding these outcomes were collected retrospectively from patient records, including surgical pathology reports, imaging reports, and clinical notes. Follow-up for survival outcomes was based on the information available in the medical records.

Statistical analysis

Continuous variables were analyzed using t-tests; categorical data, with chi-square tests. OS and DFS were estimated using the Kaplan-Meier method and compared via the log-rank test. A p-value <0.05 was considered statistically significant. IBM SPSS Statistics for Windows, Version 25 (released 2017; IBM Corp., Armonk, NY, USA) was used.

## Results

Characteristics of patients

A total of 120 patients with muscle-invasive urothelial carcinoma of the bladder (clinical stage T2-T4aN0M0), who received NAC followed by RC, were included in this retrospective analysis. There were 63 patients who received GC, with 57 receiving ddMVAC. Baseline demographic and clinical characteristics were comparable across both groups. The median age at diagnosis was 61 years in the GC group and 59 years in the ddMVAC group. Males represented 50/63 patients (79.36%) and 43/57 patients (75.43%) of the GC and ddMVAC groups, respectively. The ECOG (Eastern Cooperative Oncology Group) performance status was 0-1 for all patients. The median creatinine clearance was 75 mL/min (range: 60-115) (Table 1).

**Table 1:** Baseline characteristics Baseline characteristics of recruited patients in both arms are shown. The data have been represented as numbers and percentages of patients. Creatinine clearance (CrCl) and/or estimated glomerular filtration rate (eGFR) were calculated using the Cockcroft-Gault formula. GC, gemcitabine and cisplatin; ddMVAC, dose-dense methotrexate, vinblastine, doxorubicin, and cisplatin; ECOG, Eastern Cooperative Oncology Group; CrCl, creatinine clearance

Characteristic	GC (n = 63)	ddMVAC (n = 57)	Test Statistic	p-value
Median Age (years)	61	59	t = 1.76	0.08
Male Sex (%)	50 (79.36%)	43 (75.43%)	χ² = 0.04	0.85
ECOG 0-1 (%)	63 (100%)	57 (100%)	-	-
Clinical Stage ≥T3 (%)	34 (54%)	33 (57.89%)	χ² = 0.10	0.75
Median CrCl (mL/min)	76	74	t = 0.83	0.41

Efficacy outcomes

Pathologic Response

**Figure 1:**
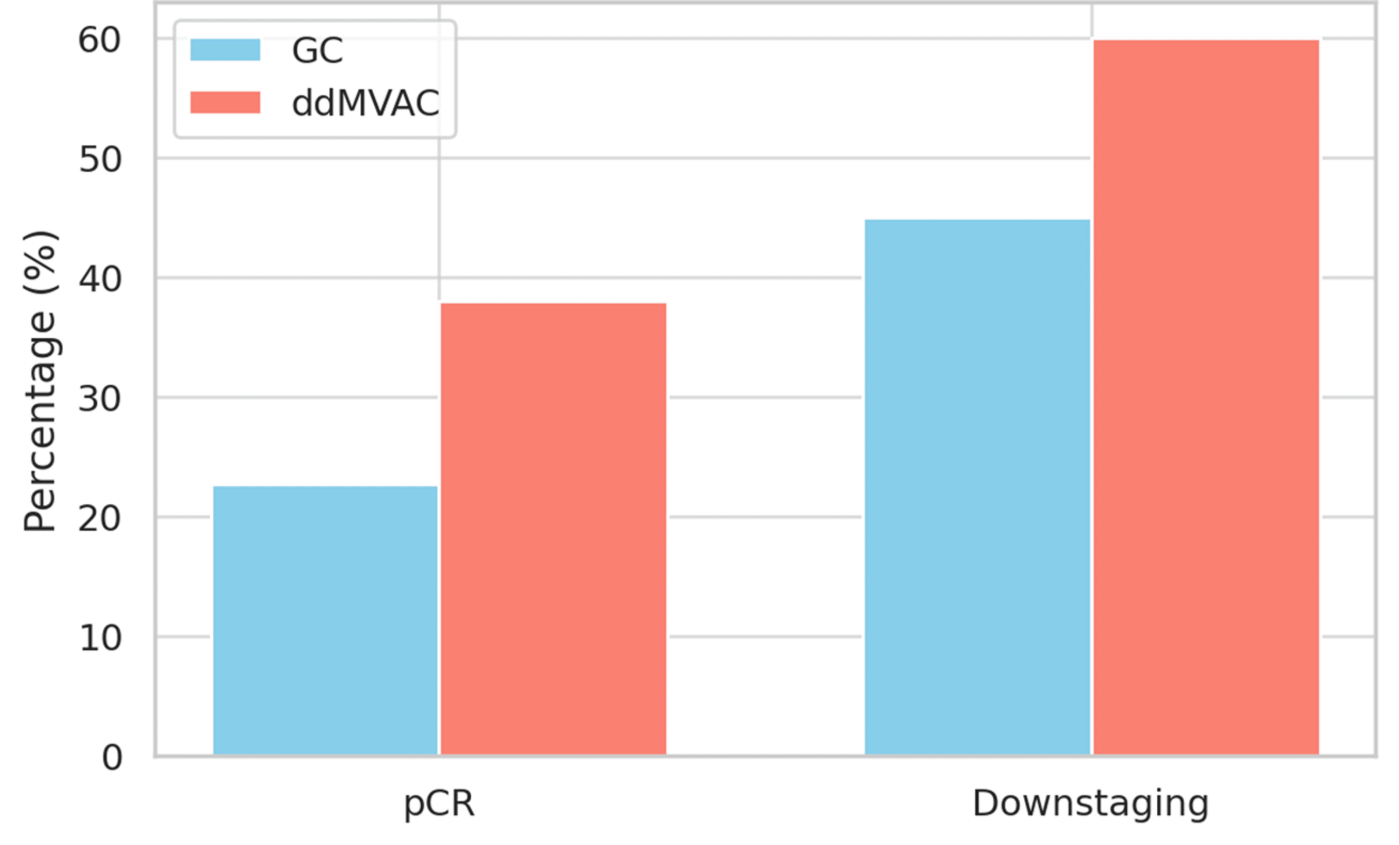
Comparison of pCR and downstaging rates This figure illustrates the comparison of pCR and downstaging rates between GC and ddMVAC. The ddMVAC regimen demonstrated a statistically significantly higher pCR rate (38.59%) compared to the GC regimen (22.22%), with a p-value of 0.04, which suggests that the observed difference in pCR rates between the two groups is statistically significant - that is, unlikely to have occurred by chance. GC, gemcitabine and cisplatin; pCR, pathological complete response; ddMVAC, dose-dense methotrexate, vinblastine, doxorubicin, and cisplatin

pCR was observed in 14 of 63 patients (22.22%) treated with GC, and in 22 of 57 patients (38.59%) treated with ddMVAC. The difference was significant (p = 0.04). Downstaging to ≤pT1N0 was seen in 28 of 63 patients (44.44%) in the GC group, and 34 of 57 patients (59.64%) in the ddMVAC group (p = 0.09) (Figure 1). 

Nodal Involvement

Residual nodal disease at the time of surgery (≥pN1) was reported in 11 of 63 patients (17.5%) in the GC group, and in 6 of 57 patients (10.5%) in the ddMVAC group. Adjuvant systemic therapy was considered for patients who did not achieve pCR or had adverse pathological features, such as ypT3/ypT4 stage or nodal positivity (ypN+).

Survival Analysis

**Figure 2:**
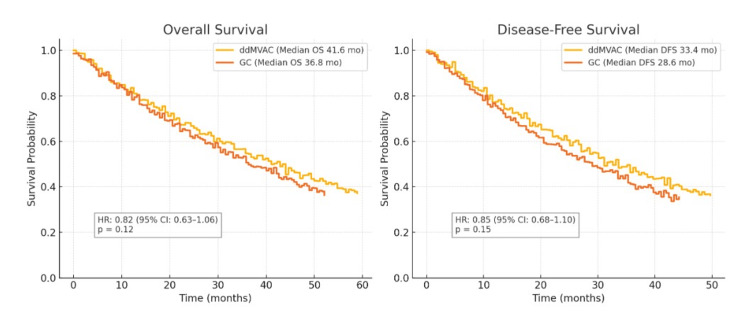
Kaplan-Meier plots for OS and DFS comparing GC to ddMVAC This figure demonstrates the Kaplan-Meier curves for OS and DFS, comparing GC to ddMVAC. Left-side image - Median OS was 41.6 months (95% CI: 35.2-48.9) in the ddMVAC group vs. 36.8 months (95% CI: 30.5-42.1) in the GC group, with p = 0.12 and HR (ddMVAC vs. GC): 0.82 (95% CI: 0.63-1.06). Right-side image - Median DFS was 33.4 months (95% CI: 27.1-39.7) for the ddMVAC group vs. 28.6 months (95% CI: 23.4-34.2) for the GC group, with p = 0.15 and HR (ddMVAC vs. GC): 0.85 (95% CI: 0.68-1.10). OS, overall survival; DFS, disease-free survival; GC, gemcitabine and cisplatin; ddMVAC, dose-dense methotrexate, vinblastine, doxorubicin, and cisplatin

With a median follow-up of 28 months (range: 12-48), the two-year DFS was 39 of 63 patients (61.9%) for GC, compared to 42 of 57 patients (73.68%) for ddMVAC. The OS at two years was 45 of 63 patients (71.42%) for GC and 46 of 57 patients (80.7%) for ddMVAC. Kaplan-Meier curves showed a divergence in favor of ddMVAC regarding DFS and OS, although the short follow-up and sample size did not allow for long-term significance. Median OS was 41.6 months (95% CI: 35.2-48.9) in the ddMVAC group vs. 36.8 months (95% CI: 30.5-42.1) in the GC group, with p = 0.12 and HR (ddMVAC vs. GC): 0.82 (95% CI: 0.63-1.06). Median DFS was 33.4 months (95% CI: 27.1-39.7) for the ddMVAC group vs. 28.6 months (95% CI: 23.4-34.2) for the GC group, with p = 0.15 and HR (ddMVAC vs. GC): 0.85 (95% CI: 0.68-1.10) (Figure 2).

Toxicity

Grade ≥3 hematologic toxicities were observed more frequently in the ddMVAC group. Neutropenia was documented in 20 of 57 patients (35%) treated with ddMVAC, compared to 10 of 63 patients (15.87%) treated with GC. Febrile neutropenia was recorded in 8 of 57 patients (14%) in the ddMVAC group vs. 3 of 63 patients (4.76%) in the GC group. Similarly, non-hematologic toxicities, such as mucositis, were more commonly documented in the ddMVAC arm, with mucositis reported in 11 of 57 patients (19.29%) compared to 3 of 63 patients (4.76%) in the GC group. Nausea/vomiting and renal toxicity showed numerically higher, but statistically insignificant, trends in the ddMVAC group. No treatment-related deaths were recorded in either cohort (Table 2). 

**Table 2:** Adverse events Important and clinically significant grade ≥3 adverse events. Data are represented as numbers and percentages of patients. GC, gemcitabine and cisplatin; ddMVAC, dose-dense methotrexate, vinblastine, doxorubicin, and cisplatin

Adverse Event	GC, N (%)	ddMVAC, N (%)
Neutropenia	10 (15.87)	20 (35)
Febrile Neutropenia	3 (4.76)	8 (14)
Mucositis	3 (4.76)	11 (19.29)
Nausea/Vomiting	8 (12.69)	11 (19.29)
Renal Toxicity	2 (3.17)	3 (5.26)

## Discussion

The best NAC regimen to treat MIBC is still being studied and debated. There is a lack of robust comparative data on GC and ddMVAC in the neoadjuvant treatment of MIBC. This single-center study does provide comparative information about the efficacy and tolerability of the two neoadjuvant regimens.

Both groups had similar median ages and baseline clinical characteristics, consistent with the demographics of patients with bladder cancer (preserved renal function, median CrCl >70 mL/min; ECOG status 0-1; male predominance). The pCR rate in the ddMVAC cohort - 22 of 57 patients (38.59%) - was significantly better than that in the GC cohort - 14 of 63 patients (22.22%) (p = 0.04). This comparison is in keeping with earlier reports (both prospective and retrospective) showing that ddMVAC had a higher pCR rate than GC. Plimack et al. reported a pCR rate of 38% with ddMVAC in a multi-institutional phase II study [8]; Zargar et al. demonstrated superior downstaging when ddMVAC was compared to GC [12].

Downstaging is a key clinical endpoint in the neoadjuvant setting, and it has significant implications for long-term oncologic outcomes. In our study, 34 of 57 patients (59.64%) receiving ddMVAC were downstaged to ≤pT1N0, compared to 28 of 63 patients (44.44%) with GC (p = 0.09), indicating that ddMVAC has a better cytoreductive effect than GC, possibly due to its higher dose intensity. The DFS and OS curves suggest a clinical benefit, based on the theory that deeper pathologic responses lead to better clinical outcomes. However, while ddMVAC is potentially superior, toxicity must be taken into account. Our retrospective data show that grade ≥3 neutropenia, febrile neutropenia, and mucositis were documented more frequently in the ddMVAC group compared to the GC group. While these toxicities were generally managed with G-CSF support and dose adjustments, the higher observed rates in the ddMVAC arm highlight the importance of careful patient selection, particularly for older individuals or those with comorbidities. Given the retrospective nature of this study, it is possible that the reported incidence of adverse events may not fully capture the actual occurrence. However, the documented trend suggests a higher burden of certain toxicities with ddMVAC, which aligns with findings from prospective studies. The comparatively lower documented hematologic toxicities in the GC group suggest a potentially better tolerability profile in our cohort, which might explain the better compliance and completion rates observed with this regimen in some patients.

These findings should be interpreted within the context of current standard-of-care recommendations. Guidelines from the NCCN and EAU recommend cisplatin-based combination chemotherapy as the treatment of choice for NAC [15]. However, to our knowledge, there is no recommendation favoring either GC or ddMVAC as the superior regimen, primarily because there are no randomized comparative trials. Though retrospective, our data enhance the growing body of literature that supports ddMVAC as a more potent regimen - when tolerated - and one that has been shown to be superior in producing complete responses and disease control.

Another aspect that warrants consideration is the utilization of immunotherapy in the perioperative setting. Studies like CheckMate 274 have shown the benefit of adjuvant nivolumab in patients with high-risk MIBC, used following NAC and cystectomy [11]. The NIAGARA trial, a phase 3 study investigating perioperative durvalumab (an anti-PD-L1 immune checkpoint inhibitor) in combination with neoadjuvant GC followed by adjuvant durvalumab monotherapy in patients with cisplatin-eligible MIBC, demonstrated significantly improved event-free and OS, as well as higher pCR rates (around 37.3% vs. 27.5% in a re-analysis). These results suggest a new standard of care. Several ongoing trials are investigating neoadjuvant enfortumab vedotin (EV), particularly in combination with immune checkpoint inhibitors, for MIBC. Trials like EV-303 (KEYNOTE-905) and various cohorts of EV-103 are exploring EV-based regimens in both cisplatin-eligible and -ineligible patients, showing promising early results and the potential to become a valuable neoadjuvant option.

Future endeavors will involve prospective randomized trials exploring GC and ddMVAC head-to-head in the neoadjuvant setting, with rigorous translational endpoints. Studies such as the SWOG S1314 (COXEN) trial [17] and Alliance A031701 [18] will attempt to do so. Additionally, biomarker-driven personalization of NAC using molecular subtypes (i.e., luminal vs. basal), genomic alterations (i.e., ERCC2 mutations), and immune signatures may improve outcomes [19,20].

In summary, this study identifies that ddMVAC can be associated with higher rates of pCR and downstaging compared to GC in the neoadjuvant management of MIBC, though with the trade-off of increased toxicity. Thus, ddMVAC can be recommended in fit patients where maximal cytoreduction is desired. In selected patients, GC still remains a valid, better-tolerated option. Ultimately, the decision for individual management of MIBC remains the same: it should be individualized based on clinical fitness, patient preference, and the emergence of new biomarkers.

Limitations of the study

It is also essential to acknowledge the shortcomings of our study. As a retrospective, single-center analysis, there is potential for selection bias, albeit both groups had similar baseline characteristics. However, treatment allocation was non-randomized, presenting possibilities of clinician or patient bias. Additional, unaccounted-for confounders - such as income, social support, nutritional factors, and the number of other coexisting illnesses - may have influenced both treatment selection and outcomes. Our study was not powered for survival endpoints, and the median follow-up was limited. Therefore, the interpretation of survival trends should be approached with caution. Toxicity findings are descriptive only and were not statistically compared, given the limitations inherent to retrospective data collection. This ensures that our conclusions remain appropriately cautious and do not overstate the findings. We were unable to analyze histologic subtypes, molecular classifications, or predictive biomarkers (which may have further stratified response to NAC), as they were not readily available for analysis.

## Conclusions

ddMVAC appears to offer better pathological response rates when compared to GC in the neoadjuvant setting of MIBC, but with the trade-off of increased toxicity. Both options are valid, and the choice should be individualized with respect to the patient’s comorbidities, renal function, and preference. Given the median follow-up time of only 28 months, the increased OS and DFS associated with ddMVAC should be viewed as sub-significant and cautiously optimistic. Future studies using prospective, randomized trial designs and biomarker-driven approaches are needed to better define patient selection.
